# Supplementary material for: Single-cell sequencing reveals the response mechanisms of vascular endothelial cells to glucocorticoids in diabetic retinopathy
Source: PLoS One. 2025 Nov 6;20(11):e0334230. doi: 10.1371/journal.pone.0334230 (PMC12591416; doi:10.1371/journal.pone.0334230)
Supplement: Attachment — Submitted filename: Response to Reviewers.docx [file pone.0334230.s010.docx]

- Response to academic editor

Concern #1: Ensure that the manuscript meets PLOS ONE's style requirements.

Author response: We have reviewed and revised our manuscript to adhere to PLOS ONE's style requirements, including file naming conventions as specified in the provided templates.

Concern #2: Code sharing guidelines.

Author response: We have ensured that all author-generated code underpinning our findings will be made available without restrictions upon publication, following the best practices outlined in PLOS ONE's code sharing guidelines. We have specified how to access the code in the data availability statement of the manuscript.

Concern #3: Data sharing restrictions.

Author response: We have uploaded all the data as supporting information files and updated the data availability statement in the manuscript accordingly.

Concern #4: Evaluate previously published works for citation.

Author response: We have reviewed the suggested publications and revised the citations in our manuscript.

We have modified the citation positions of the following literature:

Licheng Sun, Ruonan Wang, Guangyi Hu, Huazhen Liu, Kangjia Lv, Yi Duan, Ning Shen, Jiali Wu, Jing Hu, Yujuan Liu, Qihuang Jin, Fang Zhang, Xun Xu. Single cell RNA sequencing (scRNA-Seq) deciphering pathological alterations in streptozotocin-induced diabetic retinas. Experimental Eye Research. Volume 210, 2021. https://doi.org/10.1016/j.exer.2021.108718.

- Response to reviewers

#1 reviewer

Concern #1: Justify the minimal sample size statistically and address potential batch effects.

Author response: The previous statement may not have been accurate. In the original literature of this dataset, the retinal cells from three healthy mice and three diabetic mice were mixed separately to prepare cell suspensions, after which single-cell sequencing was performed. That is to say, the original statement suggested that each group corresponded to one pooled sample. In fact, each group included retinal samples from three mice, with a total of six samples. The statistical unit for single-cell sequencing is the cell rather than the sample. A total of 5,818 high-quality cells were included in this study. Through rigorous quality control, batch effect correction, and multi-dimensional analysis, an in-depth characterization of intercellular heterogeneity and subpopulation characteristics was achieved. This data-driven approach, which uses cells as the analysis unit, has been widely applied and recognized in numerous authoritative literatures, and it has strong applicability and explanatory power in exploring cell state transitions, changes in signaling pathways, and potential targets. We have adjusted the text to make the description more accurate.

Concern #2: The AUC threshold of 0.13 for GC-active cells lacks biological validation.

Author response: In this study, the AUC threshold for GC-active cells was determined based on a comprehensive analysis of the distribution of single-cell gene set enrichment scores using the AUCell tool, and methodologically, it strictly follows the data-driven grouping approach recognized in the current field. We employed the AUCell_exploreThresholds function to visualize the AUC distribution and identify inflection points. The threshold of AUC > 0.13 was automatically recommended by the AUCell_exploreThresholds function according to the AUC distribution of all cells, and was used to distinguish cell populations with high or low gene set activity. This method has been widely adopted in relevant high-level literature and gained recognition in this field（PMID: 32561888）. The current research stage is mainly data-driven, and in the future, when conditions permit, we will combine more experimental methods to further verify the biological significance of this threshold.

Concern #3: Key findings rely solely on bioinformatics and need in vitro and in vivo validation.

Author response: In this study, single-cell transcriptome sequencing data were used to systematically reveal the cell-specific roles of glucocorticoid-related signals in diabetic retinopathy (DR). Additionally, the potential functional regulatory networks of the KLF4-hydroxyurea interaction, the PTN/ANGPTL pathway, and novel hub genes such as DUSP6, AP1S2, and PTPRB were identified. These findings provide a new theoretical basis and target clues for understanding the molecular mechanism of DR and developing drug interventions.We fully acknowledge your suggestions that the biological significance of bioinformatics prediction results needs to be further confirmed through in vitro and in vivo experiments. Therefore, in the Results section, we have supplemented the qPCR validation of DUSP6, AP1S2, and PTPRB to enrich the conclusions of this study. For specific details, please refer to Figure 9. Thank you for your valuable suggestions!

Concern #4: Fig. 4F heatmap labels are illegible; Fig. 6E ligand-receptor pairs are unclear.

Author response: The layout of Figures 4 and 6 is the result of considering both the aesthetics and clarity of the figures. Reviewer 3 has raised relevant questions about some specific ligand-receptor pairs in Figure 6; therefore, we consider that the text in the figure is clearly visible. However, if the editor believes that our figures may have an impact on readers' reading, we will make further modifications to comply with the standards. Herein, we first add Fig. 4F and Fig. 6E to the supplementary materials for your reference. Thank you for pointing out the potential issues in our figures.

Concern #5: ApoE’s contradictory roles in DR need nuanced discussion.

Author response: We have expanded the discussion on ApoE's roles in diabetic retinopathy to provide a more nuanced perspective.

ApoE, or Apolipoprotein E, is a gene integral to lipid metabolism and has been extensively studied concerning diabetic complications. While some investigations have not established a robust association between ApoE polymorphisms and DR in individuals with Type 1 diabetes, other studies indicate that specific ApoE alleles might influence the severity of DR. For instance, carriers of the ApoE4 allele have been associated with a decreased risk of retinopathy in Type 2 diabetics . Furthermore, research indicates that ApoE expression is elevated in the retina when exposed to diminished light conditions, which helps alleviate vascular alterations and gliosis in diabetic retinas, pointing to a potential protective function in the progression of DR . These findings highlight the complexity of ApoE's involvement in DR and underscore the importance of considering genetic background and environmental factors in understanding its role.

→

ApoE, or Apolipoprotein E, is a gene integral to lipid metabolism and has been extensively studied concerning diabetic complications. Each of the three primary alleles—ε2, ε3, and ε4—encodes ApoE2, ApoE3, and ApoE4, respectively, and has unique roles and correlations with various diseases. Plasma ApoE protein levels are a risk factor for the development and severity of DR, according to the majority of clinical research. Moreover, genetic predisposition to DR is unaffected by polymorphisms in the ApoE gene. Nonetheless, some research indicates that particular ApoE alleles might affect how severe DR is. For instance, in the Czech population, carriers of the ε4 allele have been associated with a decreased risk of retinopathy in Type 2 diabetics. Carriers of the ε2 allele are linked to a higher risk of DR among Brazilians. DR individuals with the ε4 allele showed a strong trend toward visual impairment and more severe retinal hard exudates, according to another study on type 2 diabetic patients in Mexico The ApoE4 subtype has no influence on retinal neovascularization, although the ApoE2 and ApoE3 subtypes do, according to cell and animal studies. According to this study, variations in ApoE subtypes may affect retinal neovascularization by controlling the function of endothelial cells. The ApoE4 subtype may have an inhibitory effect in contrast to ApoE2 and ApoE3, which is in line with the findings of clinical studies. Variations in population genetic backgrounds and observational measures may be the cause of differences in study outcomes, requiring more research on the functional variability of ApoE isoforms.

#2 reviewer

Concern #1: Extremely limited sample size raises concerns about robustness and generalizability of findings.

Author response: The previous statement may not have been accurate. In the original literature of this dataset, the retinal cells from three healthy mice and three diabetic mice were mixed separately to prepare cell suspensions, after which single-cell sequencing was performed. That is to say, the original statement suggested that each group corresponded to one pooled sample. In fact, each group included retinal samples from three mice, with a total of six samples. The statistical unit for single-cell sequencing is the cell rather than the sample. A total of 5,818 high-quality cells were included in this study. Through rigorous quality control, batch effect correction, and multi-dimensional analysis, an in-depth characterization of intercellular heterogeneity and subpopulation characteristics was achieved. This data-driven approach, which uses cells as the analysis unit, has been widely applied and recognized in numerous authoritative literatures, and it has strong applicability and explanatory power in exploring cell state transitions, changes in signaling pathways, and potential targets. We have adjusted the text to make the description more accurate.

Concern #2: No validation using independent datasets or wet-lab experiments provided for proposed genes and drugs.

Author response: We fully acknowledge your suggestions that the biological significance of bioinformatics prediction results needs to be further confirmed through in vitro and in vivo experiments. Therefore, in the Results section, we have supplemented the qPCR validation of DUSP6, AP1S2, and PTPRB to enrich the conclusions of this study. For specific details, please refer to Figure 9. Thank you for your valuable suggestions!

Concern #3: Use of CellChatDB.human for mouse scRNA-seq data without justification.

Author response: Thank you for the reviewers' attention to the methodology of this study. In the cell communication analysis of this study, CellChatDB.human was selected as the reference database, mainly because it is superior to CellChatDB.mouse in terms of the completeness of ligand-receptor interaction annotations and pathway coverage, especially in core signaling pathways such as angiogenesis and immune regulation, where the information is more comprehensive. The CellChat development team also pointed out in the original literature that the core ligand-receptor interactions among different species are highly conserved, and cross-species analysis can be achieved through homologous gene mapping. Considering that the number of ligand-receptor entries in CellChatDB.mouse is relatively limited, to avoid missing potential important signals, we performed homologous matching between the human database and mouse genes before the analysis, and only retained the interaction pairs with clear corresponding relationships in the mouse annotations.

Concern #4: Some functional conclusions are speculative and require more accurate interpretations.

Author response: Following your suggestions, we have revised the statements regarding the involvement of VECs in visual perception to make the sentences more speculative rather than absolute. We have further clarified the relevant expressions as preliminary observations and emphasized that the related conclusions of this study are mainly based on single-cell transcriptome data and bioinformatics analysis. Currently, it only suggests that VECs may be involved in related pathways, and their specific physiological functions need to be further verified by subsequent experiments.

Concern #5: Clarification needed on FDR correction and multiple testing adjustments.

Author response: In this study, FDR correction was applied in the procedures of differential expression analysis, enrichment analysis, and related significance statistical processes, with FDR or q-value adopted as the significance criteria. For the AUCell scoring procedure, corresponding treatments were implemented based on its analytical characteristics. The reason for conducting multiple hypothesis test correction lies in the fact that each of our p-values is calculated through a single hypothesis test. We control the α value, that is, the probability of Type I error (the probability of incorrectly rejecting the null hypothesis), and compare it with the p-value. When the p-value is less than the α value, we reject the null hypothesis under the threshold error probability. To manage this error probability, we perform multiple hypothesis test correction to reduce false positives. However, in practice, the results of hypothesis tests fully conform to statistical principles, and not all hypothesis test results necessarily require correction. Forcing multiple corrections may instead lead to over-conservatism (for example, no significant results after correction) and mask real biological signals. Therefore, using the p-value to judge significance is sufficient （PMID: 39923360）. The detailed explanation is as follows:

Differential expression analysis was performed using the limma R package, with the screening criteria set as |logFC| > 0.25 and P value < 0.05. GO and KEGG enrichment analyses were conducted using the clusterProfiler R package, and the significance level was set at P < 0.05. The BEAM branch analysis in Monocle 2 was judged using the q-value (FDR-corrected P value) output by default in the software. The threshold for AUCell gene set activity scoring was automatically determined by the AUCell_exploreThresholds function based on the AUC distribution. Since no significance test was involved, no multiple correction was performed. The determination of significance for CellChat, related network analyses, and correlation analyses all followed the recommendations and default parameter settings of the respective R packages or software. Except for the above-mentioned analyses that did not require hypothesis testing or multiple correction, FDR correction was applied in all other cases involving multiple testing to control the false positive rate. P value < 0.05 was considered statistically significant.

Concern #6: Explanation needed for the threshold defining “glucocorticoid-active” cells.

Author response: In this study, the AUC threshold for GC-active cells was determined based on a comprehensive analysis of the distribution of single-cell gene set enrichment scores using the AUCell tool, and methodologically, it strictly follows the data-driven grouping approach recognized in the current field. The threshold of AUC > 0.13 was automatically recommended by the AUCell_exploreThresholds function according to the AUC distribution of all cells, and was used to distinguish cell populations with high or low gene set activity. We have revised the relevant descriptions in the methods and results sections to enhance readers' understanding of our analytical approach.

130 glucocorticoid-active cells were identified by establishing an ideal threshold, where cell populations with an area AUC value greater than 0.13 were categorized as having high glucocorticoid activity, and those with values less than 0.13 were classified as having low glucocorticoid activity (Fig 3A).

→

130 glucocorticoid-active cells were identified by the defined threshold, where cell populations with an area AUC value greater than 0.13 were categorized as having high glucocorticoid activity, and those with values less than 0.13 were classified as having low glucocorticoid activity (Fig 3A).

Concern #7: Clustering resolution and sensitivity testing not mentioned.

Author response: The resolution parameter of 0.5 was chosen because it yielded clustering results consistent with known retinal cell types and provided clear separation of biologically meaningful populations in our dataset. This value is commonly used in single-cell RNA-seq studies and has been demonstrated in previous literature to deliver reliable results for datasets with similar complexity and size. Therefore, we selected this resolution to balance clustering granularity and interpretability. Thank you for your valuable suggestions.

Concern #8: Gene names should be indicated in italics.

Author response: We have standardized the writing format of gene names in the revised manuscript and further improved the accuracy and clarity of the manuscript's expression. We have ensured that all gene names throughout the manuscript are now italicized, following standard conventions.

#3 reviewer

Concern #1: Clarify the sample size discrepancy between this manuscript and the original dataset.

Author response: Regarding the issues concerning the sample size and the inconsistency with the description of the original GSE178121 dataset, all the data used in our actual analysis are derived from the public expression matrix files in the GEO database. The previous statement may not have been accurate. In the original literature of this dataset, the retinal cells from three healthy mice and three diabetic mice were mixed separately to prepare cell suspensions, after which single-cell sequencing was performed. That is to say, the original statement suggested that each group corresponded to one pooled sample. In fact, each group included retinal samples from three mice, with a total of six samples. The 5,818 high-quality cells included in this study were obtained through rigorous quality control, enabling an in-depth characterization of intercellular heterogeneity and subpopulation characteristics. Through rigorous quality control, batch effect correction, and multi-dimensional analysis, an in-depth characterization of intercellular heterogeneity and subpopulation characteristics was achieved. This data-driven approach, which uses cells as the analysis unit, has been widely applied and recognized in numerous authoritative literatures, and it has strong applicability and explanatory power in exploring cell state transitions, changes in signaling pathways, and potential targets. We have adjusted the text to make the description more accurate.

Concern #2: Inaccuracy in the statement about minimal changes before and after the onset of DR.

Author response: Thank you for your careful review and valuable comments. Based on your comments and the results of data analysis, we have corrected this statement in the manuscript and provided a more accurate and objective summary of the description of the proportional changes in various cell types.

Figure 2D depicts the proportional distribution of various cell types within each sample, revealing that the majority of cells exhibited minimal changes before and after the onset of DR.

→

Fig 2D depicts the proportional distribution of various cell types in the DR group and the Control group, revealing that there may be certain changes in different cell types before and after the onset of DR.

Concern #3: Clarification on AUCell analysis and rationale for AUC cutoff.

Author response: In this study, all single-cell samples (including those from the DR group and the control group) were included in the AUCell analysis to comprehensively evaluate the activity distribution of the glucocorticoid-related gene set. The input genes were sorted in descending order based on their raw expression levels in each single cell, reflecting the enrichment of highly expressed genes in each cell. The threshold of AUC > 0.13 was automatically recommended by the AUCell_exploreThresholds function according to the AUC distribution of all cells, and was used to distinguish cell populations with high or low gene set activity. The dashed line in Figure 3A represents the boundary line automatically generated by AUCell during the threshold exploration process, and relevant information can be referred to in the official document: https://bioc.r-universe.dev/AUCell/AUCell.pdf. We have revised the relevant descriptions in the methods and results sections to enhance readers' understanding of our analytical approach.

Based on the selected 363 glucocorticoid-related genes (Table S1), high expression of glucocorticoid-related genes per cell was generated, and cells expressing more glucocorticoid-related genes had higher area under the curve (AUC) values. The "AUCell_exploreThresholds" function was used to find an appropriate threshold in order to detect active cells within the gene collection.

→

Based on the area under the curve (AUC) values of the 363 selected glucocorticoid-related genes (Table S1), the gene expression ranking for each cell was generated (sorted in descending order based on the raw expression levels within each cell) to estimate the proportion of highly expressed gene sets in each cell. Cells that express more genes in the gene set have higher AUC values. The "AUCell_exploreThresholds" function was used to determine an appropriate threshold in order to detect active cells within the gene collection.

Concern #4: Clarification on glucocorticoid-related genes in Table S3.

Author response: After taking the intersection of glucocorticoid-related genes and DEGs, we identified a total of 4 overlapping genes, namely ANGPTL4, TXNIP, PLK2, and MAPK3. Future basic research can focus on the interaction between glucocorticoids and these four genes in the context of DR. Thank you for your attention to details.

Concern #5: Difference in “Average Expression” values between Figures 5A and 5B.

Author response: Figure 5A displays the average expression levels of transcription factors with significant differences in VECs after group comparison, aiming to highlight the expression changes of these transcription factors between different groups (the DR group and the Control group). In contrast, Figure 5B reflects the overall average expression levels of these 11 transcription factors in all VECs, without conducting group comparison. The "Average Expression" values in the two figures correspond to different levels in terms of group comparison and overall statistics, respectively.

Concern #6: Clarification on transcription factors' expression patterns in Figures 5A and 5C.

Author response: Thank you for your careful review and valuable comments. We would like to clarify that Figure 5C shows the expression distribution of transcription factors in all VECs (including those in the DR group and the control group), rather than only in VECs of the DR group. Regarding the ambiguity in the description of the figure legend, we have made revisions in both the main text of the results and the legend section of the manuscript to emphasize that it refers to all VECs. Concerning the issue you pointed out about the inconsistent expression trends of some transcription factors between Figure 5A and Figure 5C, the main reason is the inconsistent grouping of the two figures. Figure 5A presents comparisons after grouping by disease status (diseased vs. normal), while Figure 5C shows the overall statistical expression levels without distinguishing between diseased and normal groups. Therefore, the trends in Figure 5A and Figure 5C are not comparable. We selected the significantly changed transcription factors mainly based on the statistical results of differential expression analysis (i.e., as shown in Figure 5A), while Figures 5B and 5C are used to visually display the expression patterns of these transcription factors in VECs. Although only some transcription factors are highly expressed in the DR group, we have presented all significantly changed transcription factors to ensure the comprehensiveness of the results. The focus of this study is to analyze the changes in transcription factors in VECs, because our analysis results indicate that VECs play an important role in the pathogenesis of DR. Although these transcription factors may also be expressed in other retinal cell types, our focus is on the specific changes and functional significance of these transcription factors in VECs. We hope that our thorough explanations will be helpful for you to gain a deeper understanding of our research.

Concern #7: More detailed discussion on ligand-receptor pairs in Figures 6B and 6C.

Author response: We have added discussions on the communication between VECs and ACs in DR, particularly the discussions on specific ligand-receptor pairs between the two types of cells, such as the Ptn-Ncl signal.

The added content is as follows:

Interestingly, we discovered that, as compared to the control group, the DR group had a higher number and strength of communication signals between VECs and ACs, as well as a substantial increase in PTN-NCL signals between VECs and ACs. ACs are key interneurons in the inner layer of the retina. The name derives from the Greek “a-” (without) and “makros” (long), referring to their lack of typical axons. According to clinical research, diabetic patients' retinal electroretinogram (ERG) shows early oscillatory potentials (OPs) alterations before the fundus examination can detect DR. OPs are considered to be the main indicator of ACs function, suggesting that ACs damage may be the earliest event in DR. In many illness models, PTN has been demonstrated to protect neurons. Following its release by VECs, PTN attaches itself to NCL on the membrane surface of ACs, perhaps halting their apoptotic process. Park et al. discovered that anti-VEGF therapy of the vitreous cavity in diabetic rats accelerated the apoptosis of ACs in the inner nuclear layer. As a result, PTN-NCL signaling may enhance ACs survival by modulating VEGF.

Concern #8: Explanation needed for decreased VEC-initiated signaling in DR progression.

Author response: Thank you very much for your concerns about the details of our article. As can be seen from Figure 6E, the listed ligand-receptor pairs do show a reduction in communication signals between VECs and other cell types. However, it should be noted that Figure 6E does not present all ligand-receptor pairs; intercellular communication signals form a large and complex network, and there are far more ligand-receptor pairs that cannot be displayed here. It can be seen from Figure 6A that compared with the Control group, the DR group shows a slight reduction in both the number and intensity of cell communications, but there may be no significant difference. This may be due to changes in the VECs environment after the onset of DR, such as damage or a downregulation of functional regulatory capacity. More studies can focus on this aspect.

Concern #9: Specify the cell population analyzed in correlation analysis.

Author response: The correlation analysis in Figure 7E was performed based on the expression data of vascular endothelial cells (VECs) identified in the single-cell transcriptome. The specific analysis workflow and the definition of cell populations have been described in the Methods section.

Concern #10: Deeper discussion of state-specific genes in the Discussion section.

Author response: Table S6 lists the specific genes corresponding to the enrichment analysis entries of different states. However, considering the large number of genes that would result in lengthy and detailed discussions that may deviate from the main topic, we did not discuss them one by one. But we have enhanced the citation of literature for reference.

Han XY, Kong LJ, Li D, Tong M, Li XM, Zhao C, Jiang Q, Yan B. Targeting endothelial glycolytic reprogramming by tsRNA-1599 for ocular anti-angiogenesis therapy. Theranostics. 2024 Jun 1;14(9):3509-3525. doi: 10.7150/thno.96946. PMID: 38948065; PMCID: PMC11209708.

Concern #11: Inquiry about transcription factors binding to hub genes.

Author response: In this study, we identified 11 transcription factors with significant changes and 25 hub genes. In Figure 8C, we constructed an mRNA-TF interaction network of hub genes through bioinformatics analysis, and further predicted that TFs are involved in the coding of hub gene mRNA. For example, Sp1 is one of the 11 transcription factors with significant changes in this study, and it is predicted in the figure to bind to genes such as NOS3 and BSG among the 25 hub genes. In fact, it has been confirmed that Sp1 can bind to the promoter region of the NOS3 gene（PMID： 25931146）. However, currently, there is limited experimental data on the direct binding of these two in the context of DR. The potential regulatory connections and subsequent mechanistic studies will become new research directions. Thank you for your valuable question.
